# Getting to Know Your Patient: Content Analysis of Patients’ Answers to a Questionnaire for Promoting Person-Centered Care

**DOI:** 10.2196/48573

**Published:** 2024-03-04

**Authors:** Juno HK Bergers, Hester Wessels-Wynia, Tatjana Seute, Astrid Janssens, Johannes JM van Delden

**Affiliations:** 1 Julius Center for Health Sciences and Primary Care University Medical Center Utrecht Utrecht Netherlands; 2 Marketing and Communication, Concern Staff University Medical Center Utrecht Utrecht Netherlands; 3 Department of Neuro-oncology University Medical Center Utrecht Utrecht Netherlands

**Keywords:** person-centered care, shared decision-making, patient engagement, positive health

## Abstract

**Background:**

Person-centered care (PCC) encourages patients to actively participate in health care, thus facilitating care that fits the life of the patient. Therefore, health care professionals (HCPs) need to know the patient. As part of a broad policy for improving PCC, a digital questionnaire (“We would like to know you”) consisting of 5 questions has previously been developed to help HCPs to get to know the patient with the help of patient and staff involvement.

**Objective:**

The purpose of this study was to provide insight into the content and aims of the questionnaire to understand its potential and usability.

**Methods:**

We conducted a qualitative, retrospective content analysis of patients’ answers using NVivo Pro (QSR International). The questionnaire was used in the outpatient neuro-oncology department of a Dutch academic hospital.

**Results:**

Of 374 invited patients, 78 (20.9%) completed the questionnaire. We selected a sample of 42 (54%) of the 78 patients. Patients used a median of 16 (IQR 7-27) words per question, and most answers were easily interpretable. When asked about important activities, social activities, sports, or maintaining a normal life were most frequently mentioned. Patients wrote about fear of the disease, its possible influence on life, or fear of the future in general. Patients wanted HCPs to know about their care and communication preferences or shared personal information. They formulated expectations about effective treatment, communication, and the care process.

**Conclusions:**

The questionnaire seems usable because patients provide interpretable answers that take little time to read, which HCPs can use to personalize care. Our study shows the potential of the questionnaire to help deliver PCC.

## Introduction

### Background

Person-centered care (PCC) is a model of care in which the active participation of patients in their own health care is encouraged. PCC is about providing holistic care to patients and not only about focusing on the patient’s disease to facilitate high-quality health care. A holistic view, taking the socioeconomic environment and psychological status into consideration, is important to obtain an overall understanding of the patient’s illness and is necessary for high-quality care [1].

Several definitions of PCC have been presented in the literature. Morgan and Yoder [2] defined PCC as follows: “PCC is a holistic (bio-psychosocial-spiritual) approach to delivering care that is respectful and individualized, allowing negotiation of care, and offering choice through a therapeutic relationship where persons are empowered to be involved in health decisions at whatever level is desired by that individual who is receiving the care.” Street [3] defines PCC using the combination of four domains: “(1) biopsychosocial approach to medical care, (2) patient as person/sharing power and responsibilities, (3) therapeutic alliance, and (4) coordinated care.” Listening to patients’ needs, values, and important topics is essential in health care. In the oath that new physicians take, they pledge to acknowledge patients’ values and needs and, in the Dutch oath especially, to listen to their patients [4,5].

Therefore, it is essential for a health care professional (HCP) to get to know the patient and to enhance communication with the patient to improve the mutual understanding of health care options and preferences. The acknowledged communication model to incorporate patients’ perspectives is shared decision-making (SDM). However, using SDM does not always mean that care is person centered. Generally, in SDM, discussing the patients’ preferences occurs after the HCP explains the available options and discusses the pros and cons [6]. SDM can result in a conversation where the HCP simply offers information and choices and cannot see the available options from the patient’s perspective [7]. Previously conducted studies have shown that health care interventions based on the patient narrative and getting to know the patient can be used to stimulate PCC in health care [8-10]. In addition, we have reason to believe that it is important to start the medical encounter by identifying what matters to the patient [11], so that the patient and the HCP together can decide which option is best in the patient’s context [7,12,13]. Barry and Edgman-Levitan [12] state that it is about teaching HCPs how to be effective partners in care. They specifically mention the potential of health care technologies that focus on better understanding patients’ experiences and eliciting patients’ needs and preferences.

In a large, Dutch academic hospital, as part of standard care in neuro-oncology, a technological initiative was introduced to facilitate PCC in daily health care. On the basis of the needs and preferences of stakeholders, patients, and HCPs of the hospital, a new, digital patient questionnaire “We would like to know you” was implemented, consisting of 5 questions. The aim of this initiative was to gather the health care preferences and needs of patients in a manner that would enable HCPs to seamlessly incorporate these needs and perspectives into medical consultations. It also aimed to provide patients the opportunity to express what they considered important for them. The initiative focused on enabling HCPs to use this information to make the consultation more receptive to patients’ contexts, needs, and preferences. Multimedia Appendix 1 shows the format of the questionnaire administered to the patients.

### Objective

An evaluation is needed to obtain information about the usefulness of this PCC tool in health care. Insight into the content of the patients’ written answers and its possible relevance for getting to know the patient is currently lacking. It is unknown whether respondents are able to answer the questions and whether these answers are interpretable. This study filled this knowledge gap by evaluating patients’ answers to the questionnaire “We would like to know you.”

## Methods

We conducted a retrospective content analysis using a qualitative, narrative research method to explore in depth the content of the questionnaire “We would like to know you” (hereafter, referred to as “the questionnaire”).

### Context

The questionnaire was developed at a large university medical center in the Netherlands. It was introduced in December 2020 as part of standard care in the outpatient neuro-oncology clinic. This department specializes in oncological diagnostics and treatment of the central nervous system.

The questionnaire was developed before commencing this study as part of a broad policy of the academic hospital to improve and facilitate PCC in daily health care practice. An internal assessment was conducted using personal interviews and a patient participation network meeting from December 2020 to April 2021. The personal interviews focused on what patients thought was important personal information to share with their HCPs. They were also asked how they wanted to share this information. HCPs answered questions about how they wanted to receive patient narratives. Overall, 21 individuals were interviewed: 10 (48%) patients and 11 (52%) HCPs. The questionnaire was further developed at a network meeting for patient participation. At this meeting, 22 members were present: 6 (27%) patients; 10 (45%) hospital employees, including HCPs; 4 (18%) students; and 2 (9%) members of the hospital’s client council. In addition, input from a neuro-oncology patient panel (n=10) was collected. Overall, 7 (70%) patients, 2 (20%) HCPs, and 1 (10%) researcher were present. All members of this panel were patients currently in treatment at that time or patients who had been treated for a neuro-oncological disease. A selection of 4 possible PCC interventions was discussed. The group decided to use the questionnaire and further discussed whether the topics of the questions and the additional information buttons were suitable for the context of the neuro-oncology.

This input was used to develop the questionnaire. No alterations to the questions were made based on the discussion.

### The Research Instrument

In this study, the questionnaire was further developed in the neuro-oncology patient panel (n=11). Overall, 8 (73%) patients or former patients, 2 (18%) HCPs, and 1 (9%) researcher participated. Again, the content of the questions and the information buttons were discussed. In addition, the format was further discussed. No alterations regarding the questions and information buttons were made. During the meeting, special attention was given to optimize the questionnaire so that it could easily be used by the patients in the clinical context and was embedded in the existing health care pathway.

This study’s questionnaire consists of 5 questions and an information button for each question. These information buttons were added to help patients answer the questions when they needed guidance. The 5 questions of the questionnaire and the content of the information buttons are presented in Table 1.

**Table 1:** Questions of the “We would like to know you” questionnaire.

Questions	Information buttons
1—What are important activities, now or in the future?	You can think of work, hobbies, or other ways you like to spend your time (traveling, sports, family and friends).
2—Which people are important in your life, and why are they important?	You can think of your partner, children, family, neighbors, friends, or people from your community, health care center, city, or other organizations.
3—What are you worried about concerning your health?	You can think of symptoms, fatigue, fear of pain, or concerns about specific things you might not be able to do in the future.
4—What do you think is important that your health care professionals know about you?	You can think of everything in relation to your care or treatment, like: do you want your doctor to address you with sir/madam or do you prefer an informal way of communication? Do you want your doctor to show pictures to explain something? Do you always want to bring a certain person to the consultation?
5—What do you expect from your treatment at the [large academic hospital]?	You can think of the results of your treatment, a regular contact person that you can always call or ask a question via e-consultations or anything else.

An internal assessment was conducted between December 2020 and April 2021. Overall, 2 HCPs of the neuro-oncology ward personally selected patients for the questionnaire based on the presumed diagnosis of a primary brain tumor and similarity of health care pathways, which included consultations with a nurse specialist, a neuro-oncologist, and a neurosurgeon. Selected patients received an invitation to answer the questionnaire together with a general introduction e-mail from the outpatient clinic before their first appointment at the hospital. From May 2021, patients were automatically selected through an electronic health record labeling system (diagnosis-treatment combination) that used the label of primary brain tumors.

The selected patients received an invitation through the hospital’s electronic personal patient portal. Patients could answer the questions on a voluntary basis, either individually or with the help of relatives, before the first hospital visit and during the entire treatment process. It was possible to answer the questions multiple times. After submission of the patient’s answers, the content of the questionnaire was accessible to HCPs involved in the patient’s care through the personal electronic health record. During internal staff meetings, the HCPs were instructed to read the patients’ answers before the consultation and were expected to address the relevant topics derived from the patients’ answers during the consultation.

### Data Collection

The data consisted of patients’ written responses to the questionnaire submitted in the period between December 2020 and August 2021. In September 2021, an HCP involved in the treatment of patients at the neuro-oncology department received a list of patients’ hospital identification numbers provided by the hospital’s IT department, which automatically registered the names of the patients who completed the questionnaire. The list consisted of patients who had started to fill in or completed the questionnaire. Because of the HCP’s involvement in treatment of the patients, the HCP had access to the electronic health records of the listed patients.

Patients’ written answers to the questionnaire were included using a sampling strategy that was based on choosing every second questionnaire on the list provided by the IT department during 3 sessions. The HCP accessed the written patient answers through the electronic health record and extracted data by pseudoanonymizing them into plain text fragments. To protect privacy, the treating HCP (TS) provided the researchers with anonymized patients’ answers, excluding information such as names, locations, and work specifications. Patients’ characteristics were collected by the treating HCP and were also presented to the researcher (JHKB).

The questionnaires were included based on their number and eligibility. They were eligible when the patients’ written answers were submitted between December 2020 and August 2021 and if the patients were still under treatment at the neuro-oncology department. The HCP did not extract written patient answers if the main treating physician was not from the neuro-oncology department. If written patient answers were not eligible, the HCP used the patient’s identification number next on the list and assessed whether the written patient answer to the questionnaire was eligible.

### Data Analysis

The aim of the analysis was to understand how patients interpreted the questions and whether their answers would help HCPs to get to know their patients Therefore, we used a content analysis approach to study the answers provided by patients [14]. We decided that the level of analysis was themes and predefined a set of categories based on the 5 questions in the survey. A researcher (JHKB) with qualitative research experience collated the answers of patients by survey question and read the answers carefully. The aim was to identify how the patients used the categories (survey questions), which would allow us to decide the usefulness and interpretability of the survey questions. Therefore, the collated answers were coded, and themes were identified. A coding tree was developed using NVivo Pro (QSR International), allowing for both deductive (predefined categories based on survey questions) and inductive codes. The inductive codes were added to reflect themes the respondents frequently addressed; they were added throughout the coding process. Practically, the first author conducted most of the work but did so in collaboration with the other authors (HWW, JJMvD, and AJ). Another researcher (HWW), skilled in narrative research, coded half of the patients’ answers independently to allow for coder triangulation. Double-coded text and the resulting coding trees were discussed, and a final tree was agreed upon. In the next step, the codes were grouped: codes were merged into existing higher-level codes, or new higher-lever codes were created to group lower-level codes. Saturation was achieved at the level of main themes. The preliminary results were also discussed with the patient panel.

The quotations used in this paper were translated into English; the original quotes were in Dutch.

### Ethical Considerations

Owing to the anonymized and retrospective nature of the study, ethics approval from the REC was not necessary according to Dutch law.

## Results

### Description of the Sample

According to the IT register, 374 patients received an invitation to complete the questionnaire between December 2020 and August 2021. Overall, 20.9% (78/374) of the patients completed the questionnaire and saved their written answers. Between December 2020 and April 2021, when patients were personally selected by HCPs for the questionnaire, 41, (41/374, 10.9%) patients received the questionnaire, 54% (22/41) answered the questions, and none (0/41, 0%) completed the questionnaire more than once.

From May 2021 to August 2021, a total of 333 (333/374, 89%) individuals were automatically provided access to the questionnaire based on a financial label of the diagnosis-treatment combination in their electronic health record. Of this group, 16.8% (56/333) of the patients completed it.

A sample of 42 (54%) written answers was selected from 78 completed questionnaires. Of this sample of 42 patients, 1 (2%) had not completed a single question, 3 (7%) answered 4 questions, and 1 (2%) answered only question 1. All 5 questions were answered by 88% (37/42) of the patients. All patients (42/42, 100%) completed the questionnaire for the first time, and none of them (0/42, 0%) completed the questionnaire more than once. The characteristics of the 42 patients are presented in Table 2. Log data were not registered by the hospital. Therefore, information about patients’ duration for completing the questionnaire, how often and for how long the information button was used, and how often and for how long an HCP looked at the questionnaire could not be collected.

**Table 2:** Patient characteristics (n=42).

Characteristics				Values, n (%)
**Sex**				
	Male		20 (48)	
	Female		22 (52)	
**Age group (y)**				
	<40		8 (19)	
	40-50		4 (10)	
	51-60		16 (38)	
	>60		14 (33)	
**Type of disease**				
	Meningioma		8 (19)	
	**Glioma**		26 (62)	
		Glioblastoma	19 (45)	
	Brain metastases		5 (12)	
	Other		2 (5)	
	Unknown		1 (2)	
Recurrence of the disease				8 (19)

### General Impression About the Written Answers

The average use of words was quite similar for all 5 questions, but there was a spread in the number of words that patients used. The numbers are presented in Table 3. Almost all patients were able to answer the questions and provided personal information. Most patients’ answers were intelligible and interpretable. In some cases, the interpretation was more difficult. For example, a person did not use punctuation, and another person seemed distrustful, possibly as a consequence of their neurological condition.

**Table 3:** Word count.

Questions	Average length of answers (words)^a^	Number of words used, median (IQR)	Spread of words^b^, range
1—What are important activities, now or in the future?	25	12 (7-29)	1-298
2—Which people are important in your life, and why are they important?	25	15 (6-24)	0-218
3—What are you worried about concerning your health?	27	17 (8-25)	0-220
4—What do you think is important that your health care professionals know about you?	33	16 (9-38)	0-280
5—What do you expect from your treatment at the [large academic hospital]?	20	17 (7-24)	0-90

^a^Values are rounded to the nearest whole number.

^b^Variation between the number of words used in the written answers.

Nearly all patients stayed close to the topic of the questions. Only in a few cases, a part of the answers entailed a topic that did not directly relate to the question and included extra, personal information. Overall, 4 (10%) of the 42 answers were elaborate, consisting of >200 words.

Most patients described things that were specific to their situation. They provided additional information, such as the name and age of their children, specific activities, personal concerns, or information about their individual situation. Some patients wrote more concisely, using general language.

### Question 1: What Are Important Activities, Now or In the Future?

When answering the first question, many people wrote about leisure activities and other social activities. Sports and family activities were mentioned most frequently. Slightly less than half of the respondents wrote about their job as an important activity. Some described driving a car or doing housework independently. A part of the respondents wrote about activities they wanted to do or keep doing in the future:

Being able to keep doing the daily housework chores including buying groceries. Exercise and cycling and going for a walk. Going on a trip with the camper (I do not drive myself). Maintaining social relationships and participating in the [organization].

Participant 18

Some patients did not mention a specific activity but wrote about ”maintaining normal activities” or “being independent.”

### Question 2: Which People Are Important in Your Life, and Why Are They Important?

The people considered to be most important were the partner, children, family, and friends. Other people mentioned were colleagues, neighbors, and other acquaintances.

More than half of the patients provided a reason why particular people were important. Respondents noted different reasons, varying from “loving the person,” “being physically and/or mentally supported by them,” or “having fun together”:

My (grand)children ([number of] sons, [number of] daughters in law, [number of] grandchildren, [number] on its way). They are my everything, I am incredibly proud of them. [They give me] support and care with lots of things, vice versa.

Participant 14

### Question 3: What Are You Worried About Concerning Your Health?

The most frequently expressed worries were about the possible influence of the disease on the patient’s life. Some patients were concerned about their health declining in general. People were worried about the development of specific physical complaints, such as brain damage, decrease in energy level, or neurological deficits. Some explicitly mentioned that they were worried about how treatment would affect their lives; others mentioned the possible influence of the disease on their loved ones. In addition, some wrote about the fear of cognitive impairment, fear of “not being themselves anymore,” or being scared to “lose control of their minds.” In addition, the influence of the disease on undertaking activities was mentioned. People worried about whether they would still be able to do their job, live independently, or stay mobile:

My disease and the uncertainty it brings. Will I be able to do my job the way I used to do it? How will the process [of working again] go? Will I be my old self regarding my energy level and will I be a nice partner for my girlfriend and a good father for my son?

Participant 7

Some worried about the disease itself. They felt that the tumor was a “thing” that was not supposed to be there and used language that referred to their disease as an entity on its own.

Some answers were about “getting better.” People were worried about whether the disease was curable and whether “everything would be alright” or that they may not have “enough time.” Furthermore, a general fear about what the future has in store was seen.

A few of the patients shared that they had no concerns, and a person explicitly did not answer the question because they wanted to stay positive. Some patients wrote the worries that they prioritized:

Physical [issues] do not bother me too much at the moment, I can cope with anything as long as I can be myself and my brain keeps working properly. Another great worry is that soon I won’t be able to function anymore and so as a freelancer I won’t have a job, income or insurance.

Participant 31

### Question 4: What Do You Think Is Important That Your HCPs Know About You?

Preferences regarding health care were frequently mentioned. An important topic was communication preferences. Respondents wrote about “clear or transparent communication” and “explaining medical information,” sometimes with pictures. Some noted that they wanted “to be addressed casually.” A number of patients wanted to take someone with them to the consultation:

I want to be addressed informally[.] I like it when people use humor and make jokes, also about my disease and treatment[.]...I want people to be honest with me and my partner about the treatment and prognosis[.] Openness and honesty is important to me.

Participant 10

In addition to discussing communication preferences, patients provided insight into their needs from and attitude toward treatment and overall health care. Some wrote that they would do anything to stay as healthy as possible, whereas a patient described the importance of having a choice:

I do not want to get every treatment, I want to think about the treatment and I want to have a choice...

Participant 3

In addition to preferences, some provided HCPs with personal information. They described how they felt, for instance, being nervous or feeling shocked by the test results. Patients also wrote about their social situation, personal characteristics, previous diseases, or current physical situation.

### Question 5: What Do You Expect From Your Treatment?

Approximately half of the patients wrote about expectations regarding their treatment goals. Some wrote about “the removal of the tumor” and the hope for “curing the disease”, or they wanted “the treatment to be effective”. A few were afraid of possible side effects:

First of all the removal of the [disease]. And that the treatment does not cause long-term harmful side effects. I don’t want to suffer from nasty side effects of a treatment like I did [number] years ago.

Participant 13

Some mentioned that they hoped to “go back to their normal lives” or wanted to “maintain quality of life.” Wishes regarding the end of life were also written. Some wanted their HCP to be professional or wanted their close ones to be involved in their care. A few specifically expected guidance from HCPs throughout the care process.

Communication was mentioned as an important topic in the answers to both questions 4 and 5. Some patients expressed their desire for “clear, transparent” communication or wrote about a regular point of contact:

It is nice to talk to the same person every time, but I am aware that this is not always possible. I always want to know what is going on with me, openness and clarity. Even if you cannot give me an answer yet, otherwise I cannot deal with it, let alone accept “it.” And yes, I want to be able to contact you for when I am having questions, it doesn’t really matter how.

Participant 14

### Information Button

Some written answers used the same words or suggested the same topics as those in the text of the information button (Table 1). For question 4, approximately one-third of the patients specifically mentioned their wish to bring someone to the consultation. Others wrote that they wanted to be addressed informally or wanted an HCP to show pictures while explaining the medical information.

Slightly less than half of the respondents wrote about the result of the treatment while answering question 5, and a few patients mentioned a regular contact person. A person seemed to directly react to the information button:

I already have a regular contact person, very nice. Pull out all the stops to get better.

Participant 9

## Discussion

### Principal Findings

This study showed that patients addressed a variety of topics related to their care. Frequently mentioned topics for important activities were leisure activities, such as social activities or sports. Some mentioned their job, and others wrote more generally about maintaining normal activities. Many respondents said that their partner, children, other family members, and friends were important people. If patients provided a reason why these people were important to them, they often wrote about loving the person or feeling supported by them. The possible influence of the disease on their health was a concern expressed most frequently by the patients. The written answers contained concerns about the effect of the disease on their physical and mental health or the effect on undertaking activities. Getting better in general was also an issue that was mentioned. For some, the tumor itself, being a thing that does not belong in someone’s head or body, made them anxious. Others wrote about fear of the future in general. The respondents wanted their HCP to know about their health care preferences, such as their treatment goals or communication preferences. Others shared information about their personal life, such as their social situation, personal characteristics, or physical situation. Expectations regarding effective treatment and the care process, including HCPs’ attitudes, the involvement of close ones, and decision-making, were emphasized.

This content appears to be relevant for health care, even if it is not always surprising. The addressed topics show similarity with the important factors regarding patients’ perception about high-quality communication [15-18]. The questionnaire provides an opportunity for patients to think about what they consider important for their health care. Moreover, it can help HCPs to follow up on these topics during the consultation. The questionnaire can be a starting point for HCPs to explore patients’ wishes, needs, and preferences relevant for a person-centered approach to care, allowing for a phenomenological approach to illness, to supplement the traditional, naturalistic medical approach.

Our results show similarities to the results of the study by Zwakman et al [19]. Zwakman et al [19] conducted a content analysis of a preference form as part of advance care planning for patients with advanced stage cancer. The preference form has questions that are comparable with questions 1, 3, 4, and 5 of this study. Both in the study by Zwakman et al [19] and in this study, maintaining a normal life and doing everyday activities were important topics. Moreover, patients valued spending time with family and friends. In both studies, patient populations expressed concerns about the effect of the treatment and the disease’s progression. Furthermore, staying independent and clear communication were important topics. However, in our study, respondents wrote less about end-of-life arrangements and alternative treatment options, possibly owing to the difference in treatment phase. Patients expressed a more precise expectation regarding their care in our study, perhaps because the question was asked more directly.

Overall, approximately 20.9% (78/374) of patients completed the questionnaire. Between December 2020 and April 2021, the percentage of completed questionnaires was higher, namely 54% (22/41). In this first phase, an internal assessment was conducted, in which the questionnaire was actively promoted. The low response percentage after April 2021 suggests that HCPs’ awareness is important and can stimulate patients to complete the questionnaire, making the questionnaire potentially more usable. Other reasons that could have influenced the response percentage were unawareness of the patient portal, difficulties in finding the questionnaire, or not wanting to answer the questions.

The selected sample showed an average word count of approximately 26 words per question, and the median number of words used was between 12 and 17. Time constraints for HCPs are a known barrier to the implementation of PCC interventions [20,21]. In this sample, patients mostly used a limited number of words, making it easy to read quickly for HCPs. Most patients were able to respond to the 5 questions and wrote an interpretable answer. Most stayed close to the topics of the questions, and some patients wrote additional information.

The information button was developed to provide guidance to patients. Our results suggest that the information buttons might influence the patients’ answers. The respondents sometimes write about particular topics mentioned in the information button, such as bringing a person to the consultation, explaining medical information with pictures, talking about a regular contact person, or having expectations about the result of treatment. Altering the text of the information button could improve the relevance of the information the questionnaire yields regarding daily health care. An example could be adjusting the information button’s text for question 4 by adding treatment preferences as a suggested topic.

### Strengths and Limitations

The strength of this study lies in the data and the thoroughness of the content analysis. Apart from the anonymized parts of the text, we used the patients’ exact words and punctuation as the treating HCP would read it. Moreover, we assessed the answers to the questionnaire regarding concise versus detailed writing and digression from and elaboration about the topic and estimated the ease of interpretation.

This study also had some limitations. First, this patient population is specific. Patients with neuro-oncological conditions have a very serious, often life-limiting disease that requires high-intensity care. It is possible that other topics found in this study may be different for other patient populations. Second, patients included in this study were affected by different histological diagnoses with therefore different prognoses and treatments for their diseases. In this study, this was not analyzed specifically. Third, our sample size of 42 patients is limited. Nevertheless, it still provides useful insights and can help to elucidate the questionnaire’s ability to improve PCC in daily health care.

### Conclusions

This questionnaire helps to stimulate patients to write about things that they consider important. By reading the patient’s answers before the consultation, HCPs can start the medical encounter with more insight into what matters to the patient. This is a major component of what determines the quality of care according to patients [22] and thus may facilitate care to become truly person centered.

This study can help the further development and implementation of the questionnaire, for instance, by adjusting the information buttons. For future studies, it may be important to repeat the questionnaire later in the care process to evaluate the possible changes in patients’ answers. The questionnaire could benefit from future studies that focus on the experiences of patients and HCPs with the questionnaire, its possible effect on the medical consultation, and the evaluation of facilitators of and barriers to its implementation in daily health care practice.

## Appendix

Multimedia Appendix 1 Format of the “We would like to know you” questionnaire administered to patients.

## Abbreviations

HCP: health care professional
PCC: person-centered care
SDM: shared decision-making

## Data Availability

The data that support the findings of this study are available from the corresponding author upon reasonable request.
