# Supplementary figures and images for: Getting to Know Your Patient: Content Analysis of Patients’ Answers to a Questionnaire for Promoting Person-Centered Care
Source: J Particip Med. 2024 Mar 4;16:e48573. doi: 10.2196/48573 (PMC10949129; doi:10.2196/48573)

Format of the “We would like to know you” questionnaire administered to patients.
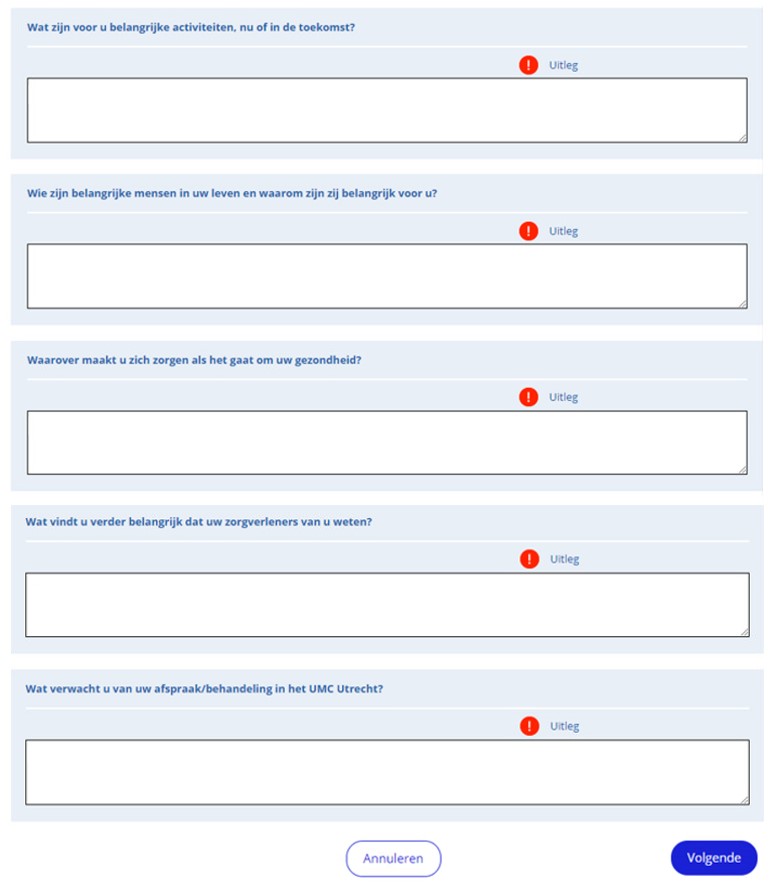

Supplement: Multimedia Appendix 1 [file jopm_v16i1e48573_app1.doc]
